# Supplementary material for: Investigating genetic links of vitamin D metabolism pathway genes (CYP2R1, CYP27B1, CYP24A1, and DBP) in Multiple Sclerosis patients
Source: PLoS One. 2025 Oct 10;20(10):e0333924. doi: 10.1371/journal.pone.0333924 (PMC12513619; doi:10.1371/journal.pone.0333924)
Supplement: S1 Fig — (DOCX) [file pone.0333924.s001.docx]

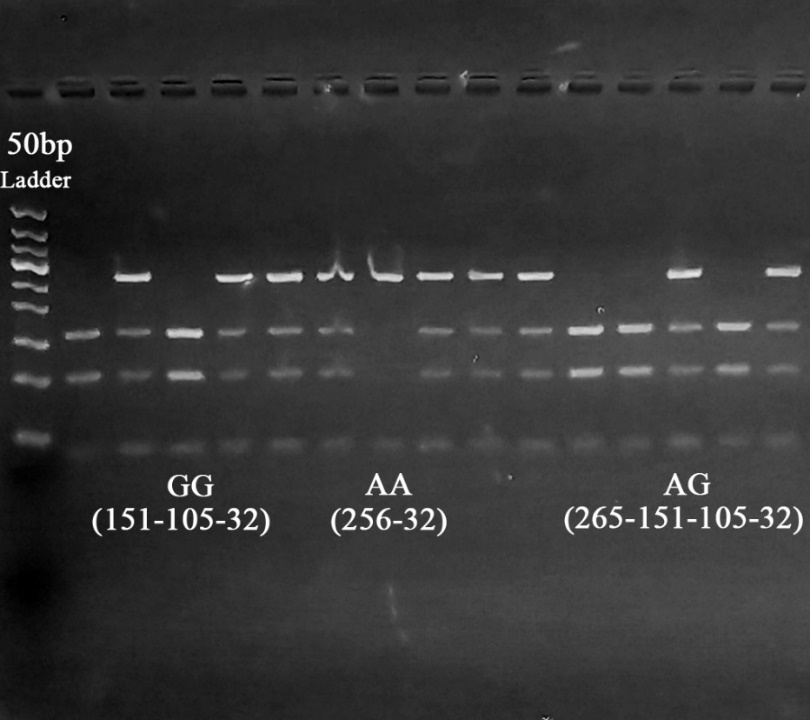


**Supplementary Figure 1.** Agarose gel electrophoresis showing different PCR-RFLP genotypes in the **CYP2R1 gene** according to SNP (rs10741657). The size of the bands was determined through comparison to a 50bp ladder. Lanes (2, 4, 5, 6, 8, 9, 10, 13, and 14) represent the heterozygous A/G genotype, with three bands at 151+105+32bp for the G/ allele and two bands at 256+32bp A/ allele; lanes (1, 3, 11, 12, and 14) contain the homozygous G/G genotype, as indicated by three bands at 151+105+32bp; while, lane (7) contain the homozygous A/A genotype, as indicated by two bands at 256+32bp
