# Supplementary material for: Investigating genetic links of vitamin D metabolism pathway genes (CYP2R1, CYP27B1, CYP24A1, and DBP) in Multiple Sclerosis patients
Source: PLoS One. 2025 Oct 10;20(10):e0333924. doi: 10.1371/journal.pone.0333924 (PMC12513619; doi:10.1371/journal.pone.0333924)
Supplement: S2 Fig — (DOCX) [file pone.0333924.s002.docx]

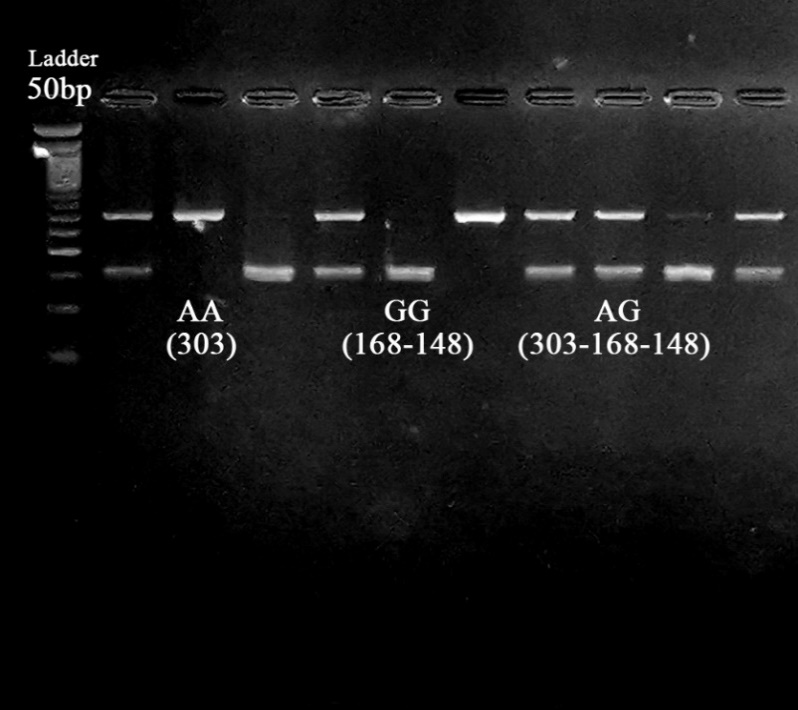


**Supplementary Figure 2.** Agarose gel electrophoresis showing different PCR-RFLP genotypes in the **CYP2R1 gene** according to SNP (rs1279714). The size of the bands was determined through comparison to a 50bp ladder. Lanes (1, 4, 7, 8, 9, and 10) represent the heterozygous A/G genotype, with two bands at 168+148bp for the G/ allele and one band at 303bp A/ allele; lanes (3 and 5) contain the homozygous G/G genotype, as indicated by two bands at 168+148bp; while, lanes (2 and 6) contain the homozygous A/A genotype, as indicated by one band at 303bp.
