# Supplementary material for: Investigating genetic links of vitamin D metabolism pathway genes (CYP2R1, CYP27B1, CYP24A1, and DBP) in Multiple Sclerosis patients
Source: PLoS One. 2025 Oct 10;20(10):e0333924. doi: 10.1371/journal.pone.0333924 (PMC12513619; doi:10.1371/journal.pone.0333924)
Supplement: S3 Fig — (DOCX) [file pone.0333924.s003.docx]

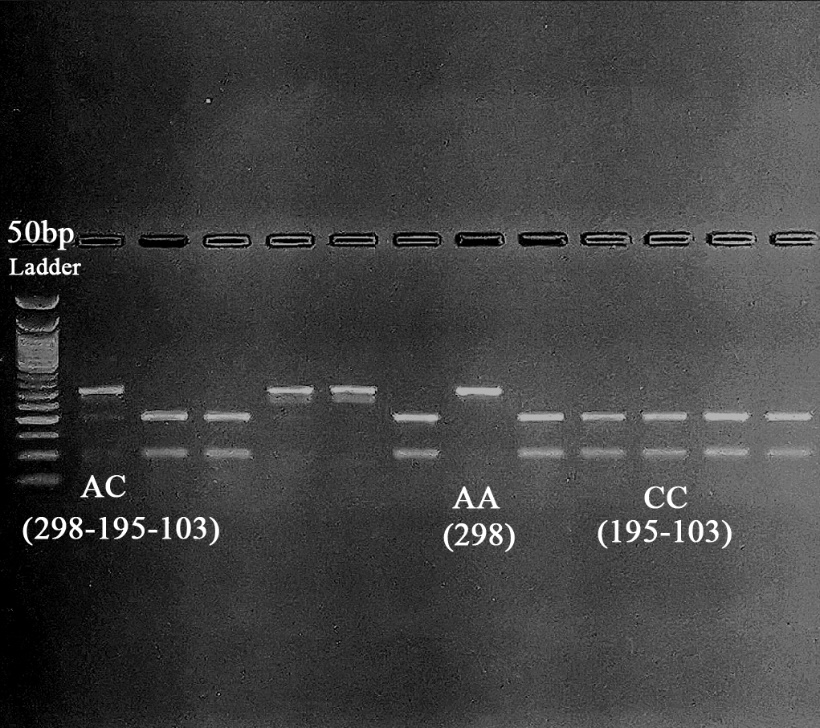


**Supplementary Figure 3.** Agarose gel electrophoresis showing different PCR-RFLP genotypes in the **CYP27B1 gene** according to SNP (rs10877012). The size of the bands was determined through comparison to a 50bp ladder. Lanes (1, 4, and 5) represent the heterozygous A/C genotype, with two bands at 195+103bp for the C/ allele and one band at 298bp A/ allele; lanes (2, 3, 6, 8, 9, 10, 11, and 12) contain the homozygous C/C genotype, as indicated by two bands at 195+103bp; while, lane (7) contain the homozygous A/A genotype, as indicated by one band at 298bp.
