# Supplementary material for: Investigating genetic links of vitamin D metabolism pathway genes (CYP2R1, CYP27B1, CYP24A1, and DBP) in Multiple Sclerosis patients
Source: PLoS One. 2025 Oct 10;20(10):e0333924. doi: 10.1371/journal.pone.0333924 (PMC12513619; doi:10.1371/journal.pone.0333924)
Supplement: S4 Fig — (DOCX) [file pone.0333924.s004.docx]

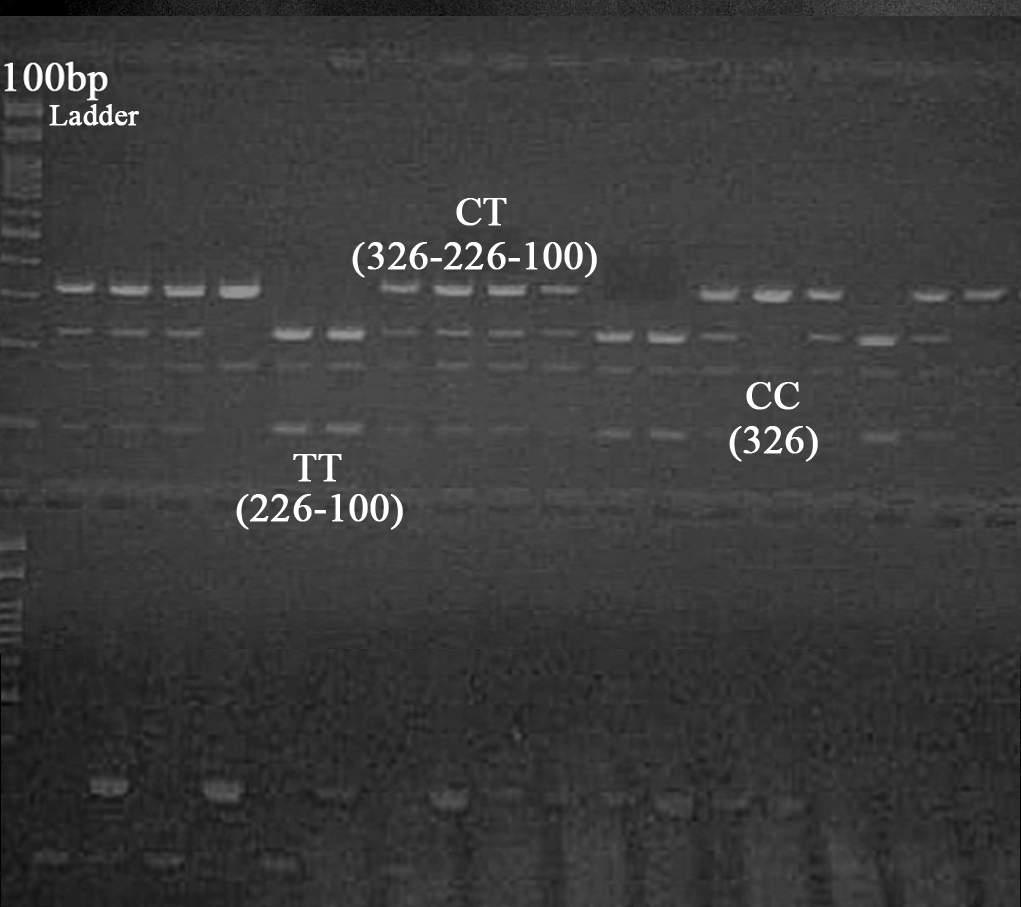


**Supplementary Figure 4.** Agarose gel electrophoresis showing different PCR-RFLP genotypes in the **CYP24A1 gene** according to SNP (rs2248359). The size of the bands was determined through comparison to a 100bp ladder. Lanes (1,2,3,7,8,9,10,13,15 and 17) represent the heterozygous C/T genotype, with two bands at 226+100bp for the T/ allele and one band at 326bp C/ allele; lanes (5,6,11,12, and 16) contain the homozygous T/T genotype, as indicated by two bands at 226+100bp; while, lane (4,14, and 18) contain the homozygous C/C genotype, as indicated by one band at 326bp.
