# Supplementary material for: Investigating genetic links of vitamin D metabolism pathway genes (CYP2R1, CYP27B1, CYP24A1, and DBP) in Multiple Sclerosis patients
Source: PLoS One. 2025 Oct 10;20(10):e0333924. doi: 10.1371/journal.pone.0333924 (PMC12513619; doi:10.1371/journal.pone.0333924)
Supplement: S5 Fig — (DOCX) [file pone.0333924.s005.docx]

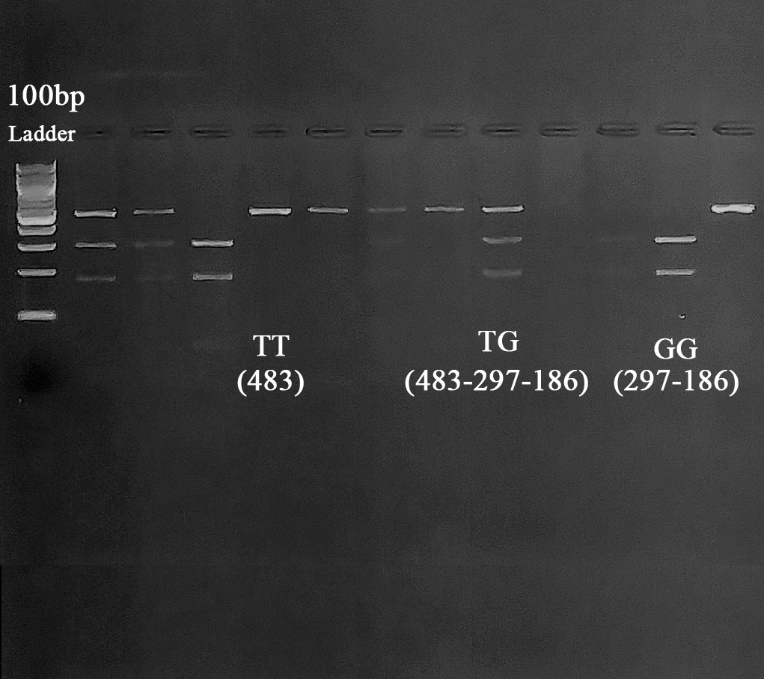


**Supplementary Figure 5.** Agarose gel electrophoresis showing different PCR-RFLP genotypes in the **DBP gene** according to SNP (rs7041). The size of the bands was determined through comparison to a 100bp ladder. Lanes (1, 2, and 8) represent the heterozygous T/G genotype, with two bands at 297+186bp for the G/ allele and one band at 483bp T/ allele; lanes (3, 10, and 11) contain the homozygous G/G genotype, as indicated by two bands at 297+186bp; while, lanes (4, 5, 6, 7, and 12) contain the homozygous T/T genotype, as indicated by one band at 483bp.
