# Supplementary material for: Investigating genetic links of vitamin D metabolism pathway genes (CYP2R1, CYP27B1, CYP24A1, and DBP) in Multiple Sclerosis patients
Source: PLoS One. 2025 Oct 10;20(10):e0333924. doi: 10.1371/journal.pone.0333924 (PMC12513619; doi:10.1371/journal.pone.0333924)
Supplement: S6 Fig — (DOCX) [file pone.0333924.s006.docx]

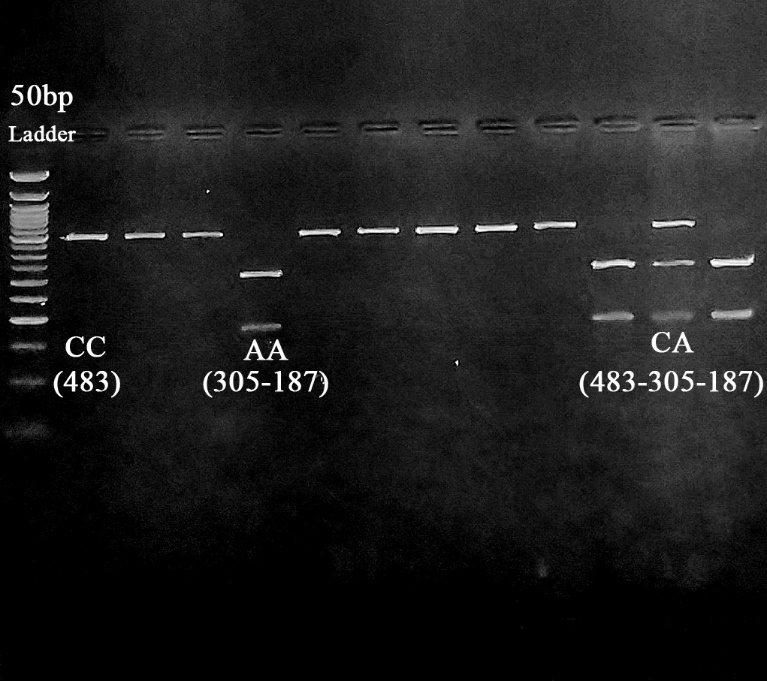


**Supplementary Figure 6.** Agarose gel electrophoresis showing different PCR-RFLP genotypes in the **DBP gene** according to SNP (rs4588). The size of the bands was determined through comparison to a 50bp ladder. Lane (11) represent the heterozygous C/A genotype, with two bands at 305+187bp for the A/ allele and one band at 483bp C/ allele; lanes (4, 10 and 12) contain the homozygous A/A genotype, as indicated by two bands at 305+187bp; while, lanes (1, 2, 3, 5, 6, 7, 8, and 9) contain the homozygous C/C genotype, as indicated by one band at 483bp.
