# Supplementary material for: Investigating genetic links of vitamin D metabolism pathway genes (CYP2R1, CYP27B1, CYP24A1, and DBP) in Multiple Sclerosis patients
Source: PLoS One. 2025 Oct 10;20(10):e0333924. doi: 10.1371/journal.pone.0333924 (PMC12513619; doi:10.1371/journal.pone.0333924)
Supplement: S1 Table — (DOCX) [file pone.0333924.s007.docx]

**Supplementary Table 1.** The sequences of forward and reverse primers used for the analysis of all SNPs

| **Gene and SNP Name** | | **Forward and Reverse Primer Sequences** | **Restriction Enzymes** | **Reference** |
| --- | --- | --- | --- | --- |
| ***CYP2R1*** | **rs10741657** | 5′-GGGAAGAGCAATGACATGGA-3′ | **MnII** | (27) |
|  |  | 5′-GCCCTGGAAGACTCATTTTG-3′ |  |  |
|  | **rs12794714** | 5’-GGAAGCTTTGGAGAGCTGAA-3’ | **FokI** | (28) |
|  |  | 5’-GCCATAAGTCCAACCAGGAA-3’ |  |  |
| ***CYP27B1*** | **rs10877012** | 5'-TTCAATTCCAGAACTTCAGAGC-3' | **TfiI** | (29) |
|  |  | 5'-AACATAGTCGAACTGTCTCTAC-3' |  |  |
| ***CYP24A1*** | **rs2248359** | 5’- AGTTAGGAAATGCGCCTTGAG-3’ | **SacII** | (30) |
|  |  | 5’- GGATCAGGTTGAAAGGATTCG-3’ |  |  |
| ***DBP*** | **rs7041** | 5′-AAATAATGAGCAAATGAAAGAAGAC-3’ | **HaeIII** | (31) |
|  |  | 5′-CAATAACAGCAAAGAAATGAGTAGA-3’ |  |  |
|  | **rs4588** | 5′-AAATAATGAGCAAATGAAAGAAGAC-3’ | **StyI** |  |
|  |  | 5′-CAATAACAGCAAAGAAATGAGTAGA-3’ |  |  |
