# Supplementary material for: Investigating genetic links of vitamin D metabolism pathway genes (CYP2R1, CYP27B1, CYP24A1, and DBP) in Multiple Sclerosis patients
Source: PLoS One. 2025 Oct 10;20(10):e0333924. doi: 10.1371/journal.pone.0333924 (PMC12513619; doi:10.1371/journal.pone.0333924)
Supplement: S2 Table — (DOCX) [file pone.0333924.s008.docx]

| **Genes** | **SNPs** | **Alleles** | **PCR program (35 cycles)** | **PCR Product Size** | **Restriction Endonuclease**  **Incubation temperature** | **Fragments Size (bp)** |
| --- | --- | --- | --- | --- | --- | --- |
| ***CYP2R1*** | **rs10741657** | **(A/G)** | **94^o^C for 3 min (94^o^C for 30s, 57^o^C for 30s, 72^o^C for 40s) 72^o^C for 7 min** | **288 bp** | **MnII / 37^o^C** | **AA**:256-32  **AG**:256-151-105-32  **GG**:151-105-32 |
|  | **rs12794714** | **(A/G)** | **95^o^C for 10 min (95^o^C for 1 min, 67^o^C for 1 min, 72^o^C for 1 min) 72^o^C for 10 min** | **303 bp** | **FokI / 37^o^C** | **AA**:303  **AG**: 303-168-148  GG: 168 - 148 |
| ***CYP27B1*** | **rs10877012** | **(A/C)** | **94^o^C for 5 min (92^o^C for 30s, 55^o^C for 45s, 72^o^C for 40s) 72^o^C for 5 min** | **298 bp** | **TfiI / 37^o^C** | **AA**:298  **AC**:298-195-103  **CC**:195-103 |
| ***CYP24A1*** | **rs2248359** | **(C/T)** | **95ºC for 5 min, (95 ºC for 1 min, 53 ºC for 2 min, 72 ºC for 1 min) 72 ºC for 3 min)** | **326 bp** | **SacII / 37oC** | **CC:**326  **CT:**326-226-100  **TT:**226-100 |
| ***DBP*** | **rs7041** | **(T/G)** | **94^o^C for 3 min (92^o^C for 30s, 55^o^C for 30s, 72^o^C for 40s) 72^o^C for 10 min** | **483 bp** | **HaeIII / 37^o^C** | **TT**:483  **TG**:483-297-186  **GG**:297-186 |
|  | **rs4588** | **(C/A)** | **94^o^C for 3 min (92^o^C for 30s, 55^o^C for 30s, 72^o^C for 40s) 72^o^C for 10 min** | **483 bp** | **StyI / 37^o^C** | **CC**:483  **CA**:483-305-187  **AA**:305-187 |

**Supplementary Table 2.** PCR product size of DNA, and restriction fragment sizes for all SNPs
